# Experimental Investigation of the Rapid Fabrication of Micron and Submicron Structures on Polymers Utilizing Ultrasonic Assisted Embossing

**DOI:** 10.3390/polym13152417

**Published:** 2021-07-23

**Authors:** Yongyong Zhu, Sebastian Bengsch, Lei Zheng, Yangyang Long, Bernhard Wilhelm Roth, Marc Christopher Wurz, Jens Twiefel, Jörg Wallaschek

**Affiliations:** 1 Institute of Dynamics and Vibration Research, Leibniz University Hannover, An der Universität 1, 30823 Garbsen, Germany; long@ids.uni-hannover.de (Y.L.); twiefel@ids.uni-hannover.de (J.T.); joerg.wallaschek@ids.uni-hannover.de (J.W.); 2 Cluster of Excellence PhoenixD (Photonics, Optics, and Engineering–Innovation Across Disciplines), Leibniz University Hannover, 30167 Hannover, Germany; 3 Institute of Micro Production Technology, Leibniz University Hannover, An der Universität 2, 30823 Garbsen, Germany; bengsch@impt.uni-hannover.de (S.B.); wurz@impt.uni-hannover.de (M.C.W.); 4 Hannover Centre for Optical Technologies, Leibniz University Hannover, Nienburger Straße 17, 30167 Hannover, Germany; lei.zheng@hot.uni-hannover.de (L.Z.); bernhard.roth@hot.uni-hannover.de (B.W.R.)

**Keywords:** ultrasonic vibration, embossing, micron and submicron structure, impedance-based control

## Abstract

Small-scale optical components with micron or submicron features have grown in popularity in recent years. High-quality, high-efficient, and cost-effective processing approaches for polymer optics mass production are an urgent need. In this study, ultrasonic vibration will be introduced in embossing. The major advantage is that the required energy can be provided for process times ranging from a few hundred milliseconds to a few seconds, and that the process energy is provided at exactly the required location so that the structures in the surrounding area are not affected. Due to the strong correlation between electrical impedance and the temperature of the material, a novel impedance-based control strategy has been utilized for precisely controlling ultrasonic vibration during the embossing process. The investigation used two types of stamps with grating line widths of 4 µm and 500 nm, respectively. As a result, an embossing time of less than a few seconds was accomplished and a uniform embossed surface with an average fill rate of more than 75% could be achieved.

## 1. Introduction

Optical components with micron or submicron structures have become increasingly popular in recent years. Fiber Bragg gratings, which are commonly used in the fields of communication and sensors, are one of the most promising applications. Hill et al. produced the first fiber Bragg gratings in 1978 [1,2]. Nowadays, two-beam interferometric [3], sequential writing [4], point by point [5], and photomask [6] are the standard manufacturing methods for fiber Bragg gratings. In recent years, optical polymer components have become more widely used, and more manufacturing methods have been improved and integrated into the production of micro- or nanometer structured polymers, the most popular of which are micro injection molding [7], micro extrusion [8], hot embossing [9,10,11], and ultraviolet curing embossing [12].

Comparing these processing methods mentioned above, ultraviolet curing embossing is the only method that does not need an external heat source. The processing temperature of micro extrusion and micro injection molding is higher than the melting temperature ($T_{m}$), while the working temperature of hot embossing is between glass transition temperature ($T_{g}$) and melting temperature ($T_{m}$). Except for micro extrusion, all of the approaches have a high level of production accuracy. However, due to the method’s limitations, micro injection molding is usually used to manufacture products with thinner thickness. This method requires high pressure during the operation and is generally only suitable for micron-scale manufacturing. Due to the high cost of raw materials and tools, ultraviolet curing embossing raises production costs. Because of its lower production cost, higher replication accuracy, and compatibility with both micro- and nanoscale manufacturing, hot embossing is ideal for batch fabrication. However, since the heating and cooling process takes 10 to 25 min [13], it has the disadvantage of low efficiency.

**Figure 1:**
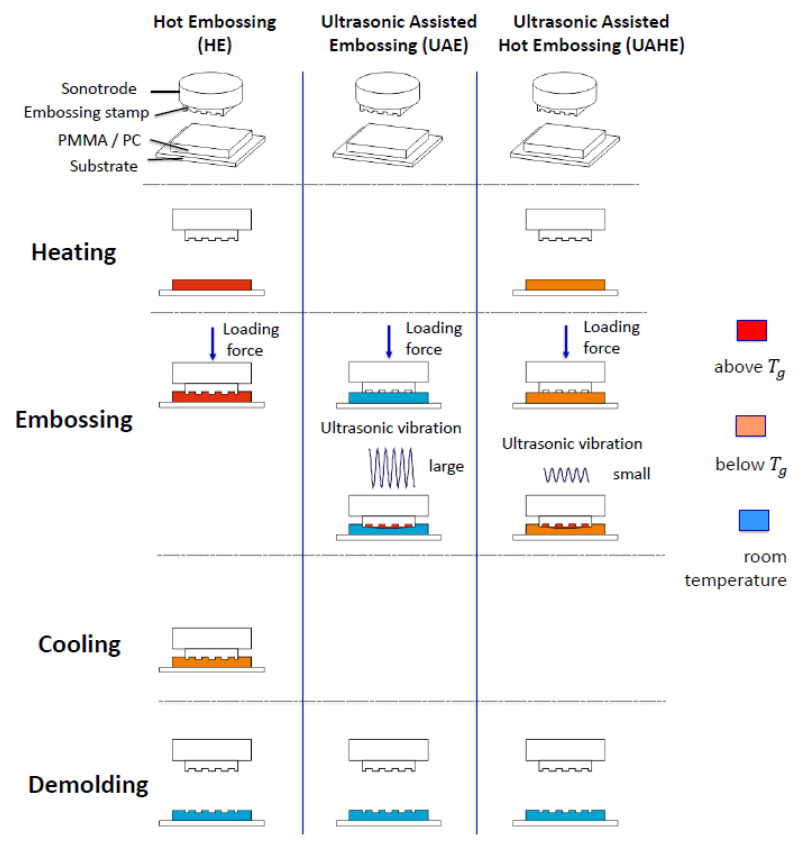
Process chart of hot embossing (HE), ultrasonic assisted embossing (UAE), and ultrasonic assisted hot embossing (UAHE).

Ultrasonic vibrations are introduced into the embossing process primarily to reduce the process time. Embossing with ultrasound can be divided into two types: ultrasonic assisted embossing (UAE) and ultrasonic assisted hot embossing (UAHE) (Figure 1).

The difference is that ultrasonic assisted embossing produces heat entirely from ultrasonic vibrations, while ultrasonic assisted hot embossing involves an additional heat source. Ultrasonic assisted manufacturing technology has been widely used for welding, cutting, and machining due to its simple and effective processing, as well as its good manufacturing quality. This article will address the use of ultrasonic assisted embossing for rapid production of micro and submicrometer structures in polymers, such as those needed for waveguides and fiber Bragg grating. Ultrasonic assisted embossing has many advantages over other methods, including a lower energy input, a smoother operation, improved embossing performance, and a higher aspect ratio.

Mechanical and optical properties are two other important indicators of embossing quality. Compared to injection molding, micro extrusion, hot embossing, and ultrasonic assisted embossing, the mechanical properties of the products manufactured by ultraviolet curing embossing are generally low. During ultrasonic-assisted embossing, the creep of the molecular chains is enhanced by ultrasonic vibrations and the flow of the melt is improved [14]. As a result, the embossed structures will have better mechanical properties. Furthermore, the vibrational energy of ultrasonic embossing is heated in localized areas, resulting in lower residual stresses. For embossed optical components, the lower residual stresses result in a more uniform refractive index within the structure, which provides better optical qualities.

For polymers, the glass transition temperature ($T_{g}$) is a significant parameter. Polymers are complicated in terms of atom structure and softening processes, and they can be brittle, elastic, or viscous depending on the temperature. Amorphous and crystalline polymers are the two types of polymers. This research will focus on amorphous polymers (polycarbonate). Polymers have different material properties at different temperatures. When the polymeric material has been heated to $T_{g}$ temperature it becomes less fluid, but still difficult to deform. It also has weaker filling properties.

Earlier studies of ultrasonic assisted embossing by other researchers are listed in Table 1. The majority of them tend to focus on structures at the millimeter or tens of micron scale [15,16,17,18,19,20,21,22], and Fan et al. [23] investigated embossing of 2 µm structures. It is more difficult to emboss thinner and deeper grating lines. Meanwhile, nearly all the control strategies, including ultrasonic vibration time and hold time, were centered on time control in the previous investigations. Compared with the time control strategy, the impedance-based control method can better adapt to different materials and molds with different geometry and stop the ultrasonic vibration more accurately, thus avoiding over-embossing. In this investigation, a simple setup for ultrasonic assisted embossing with impedance-based control strategy was used to emboss the mold’s structure with several microns and hundreds of nanometers, in order to understand the potential of this processing and make it possible to mass-produce or integrate it in a roll-to-roll (R2R) or roll-to-plate (R2P) process.

## 2. Experimental Setup

### 2.1. Test Bench

**Figure 2:**
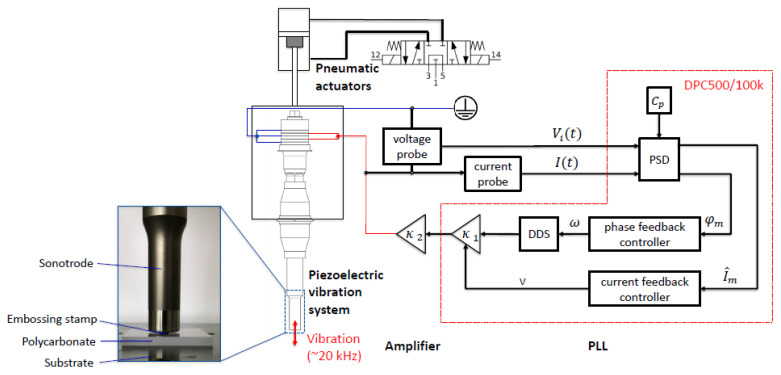
Schematic diagram of the ultrasonic assisted embossing system. PSD: phase sensitive detector; DDS: direct digital synthesis.

The ultrasonic assisted embossing setup consisted of three systems, including an ultrasonic vibration system, a control system, and a feeding system. The core of the ultrasonic vibration system was an ultrasonic transducer whose first longitudinal vibration mode was at approximately 20 kHz. The frequency of 20 kHz is usually applied for power ultrasonics. This is due to the fact that this frequency is beyond the human ear’s audible range. As a result, vibration-induced noise is considerably decreased. Second, the higher the frequency is, the lower the ultrasonic power intensity that can be reached. For embossing with a large stamp, a higher ultrasonic power intensity is desired. The embossing mold was attached to the end of the transducer. The transducer was driven by an in-house built DPC500/100K controller [24] and a QSC5050 power amplifier. The DPC500/100K controller had two major control functions: phase feedback control and current feedback control (the red box in Figure 2). The pneumatic device (feeding system) controlled the transducer’s vertical movement and provided a constant loading force during the embossing process. The whole experimental setup is schematically shown in Figure 2.

The phase between the voltage and current of the transducer was set to zero. This enabled the transducer to run at its resonant frequency and to generate vibration efficiently. As the transducer was operated at its resonant frequency, the transducer’s vibration amplitude was proportional to the current amplitude. Thus, setting the reference current allowed us to control the transducer’s vibration amplitude.

### 2.2. Embossing Pattern

**Figure 3:**
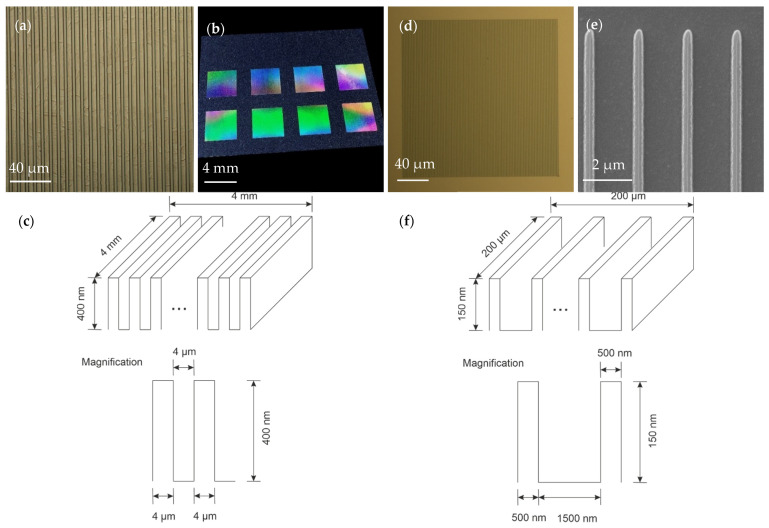
(**a**) 4 µm gratings on steel stamp; (**b**) diffraction behavior of 4 µm gratings–stamp; (**c**) schematic diagram of embossing pattern with 4 µm grating; (**d**) 500 nm gratings on steel stamp; (**e**) 500 nm gratings on steel stamp (SEM-Image); (**f**) schematic diagram of embossing pattern with 500 nm grating.

Two different sizes of embossing stamps with Bragg grating structures were constructed to investigate the structure’s transfer to the polymer substrate. UV lithography and lift-off techniques were used to manufacture the micron-scale structure with a footprint of 4 × 4 mm^2^ (in Figure 3a–c). The 500 nm gratings shown in Figure 3d–f were structured using e-beam lithography and had a footprint of 200 × 200 µm^2^. As a thin film material, permalloy was used. For the lift-off, a photoresist (AZ5214 by MicroChemicals GmbH, Germany) was employed to build the grating structures.

### 2.3. Procedure of Ultrasonic Assisted Embossing

**Figure 4:**
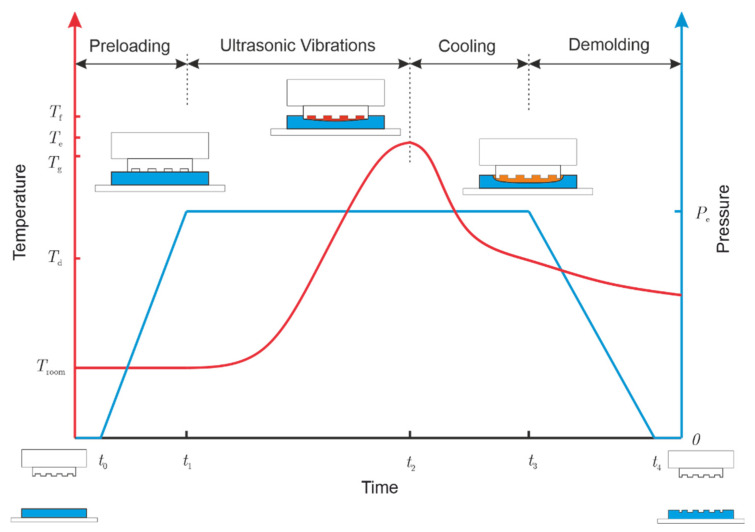
Procedure of ultrasonic assisted embossing.

The ultrasonic assisted embossing process is shown in Figure 4. In comparison to traditional hot embossing, ultrasonic assisted embossing does not require a preheating step. After the preloading of the stamp, the ultrasonic vibrations were activated. During the vibration period, the temperature increased rapidly until it reached the embossing temperature ($T_{e}$), which was 20–30 °C higher than the glass transition temperature ($T_{g}$) and lower than the flow temperature ($T_{f}$). Once the ultrasonic vibrations stopped, a cooling stage followed and the temperature dropped. Finally, the loading force was released in the demolding stage. Since no external heat was needed and the ultrasonic energy was mainly absorbed within the embossing region instead of the whole substrate, ultrasonic assisted embossing consumed low energy and created structures efficiently.

## 3. Preliminary Study of the Setup

**Figure 5:**
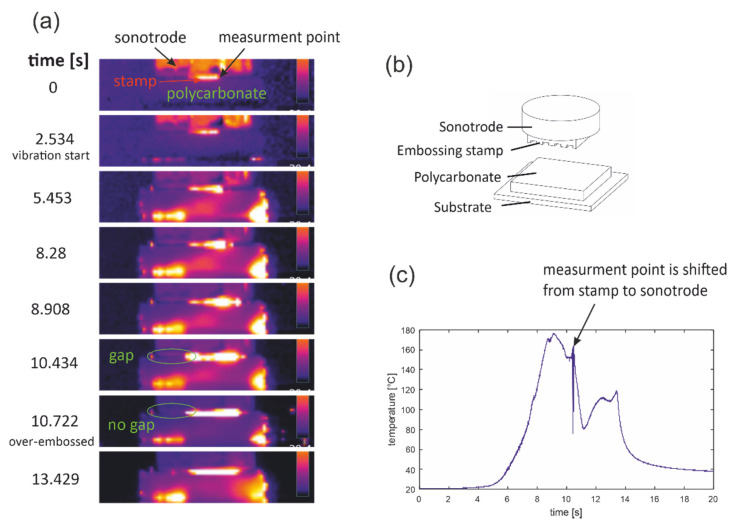
(**a**) Thermal imaging of embossing process with thermal camera; (**b**) schematic diagram of imprinted components; (**c**) temperature curve (the measurement point is showed in (**a**)).

Temperature is an important factor in embossing polymer materials. The analysis was conducted with a smaller amplitude (current: 2.7 A, vibration amplitude: 20 μm). To monitor the temperature changes during the entire operation, a FILR thermal imaging camera (FLIR SC7300) was used. As shown in Figure 5, the temperature started to increase at 2.5 s when the ultrasound was activated. The temperature rose sharply between 6 and 8 s, from approximately 40 to 140 °C (see Figure 5c), which is close to polycarbonate’s glass transition temperature ($T_{g}$). At around 9 s, the embossing region was heated to 20–30 °C above $T_{g}$ temperature, at which the polycarbonate in the contact region entered a rubbery state. The structures could then be transferred to the plastic substrate. This can be also observed in Figure 5a. At 10.434 s, a gap still existed between the transducer and the polymer, but after 0.3 s, the gap vanished, showing that the embossing pattern was fully embedded in the polymer. The temperature then dropped abruptly. At the same time, due to the deformation of the polymer substrate, the measurement point shifted to the sonotrode.

The polymer material progressively transformed from glassy to viscoelastic as the temperature rose from room temperature to $T_{g}$ temperature, and the material’s damping increased. This was reflected in the ultrasonic vibration system by a higher impedance of load. The impedance $Z_{L}$ can be determined by the following equations:

(1) $$Z_{L}=\frac{U}{I}(\cos\varphi +i\sin\varphi )$$

(2) $$R=\frac{U}{I}\cos\varphi$$

(3) $$\left|Z_{L}\right|\approx \left|R\right|=\frac{\hat{U}}{\hat{I}}\;\left(when\;\varphi \leq 5{}^{\circ}\right)$$

where U represents voltage, I represents current, and φ is the phase difference between voltage and current.

**Figure 6:**
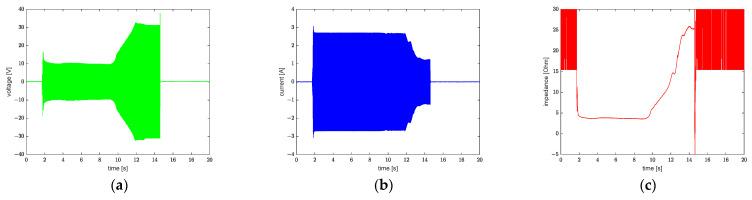
(**a**) Voltage curve; (**b**) Current curve; (**c**) Impedance curve.

The phase between the voltage and current of the transducer is minimal since it operates at the resonant frequency, therefore the impedance can be determined from the voltage and current amplitude (Equation (3)). At ~10 s, the impedance ($Z_{L}$) began to increase from its previous value (in Figure 6), which remained basically unchanged, indicating that the pattern was then capable of being embossed into the polycarbonate substrate. The impedance is highly correlated with temperature, as can be seen from Figure 5c and Figure 6c.

**Figure 7:**
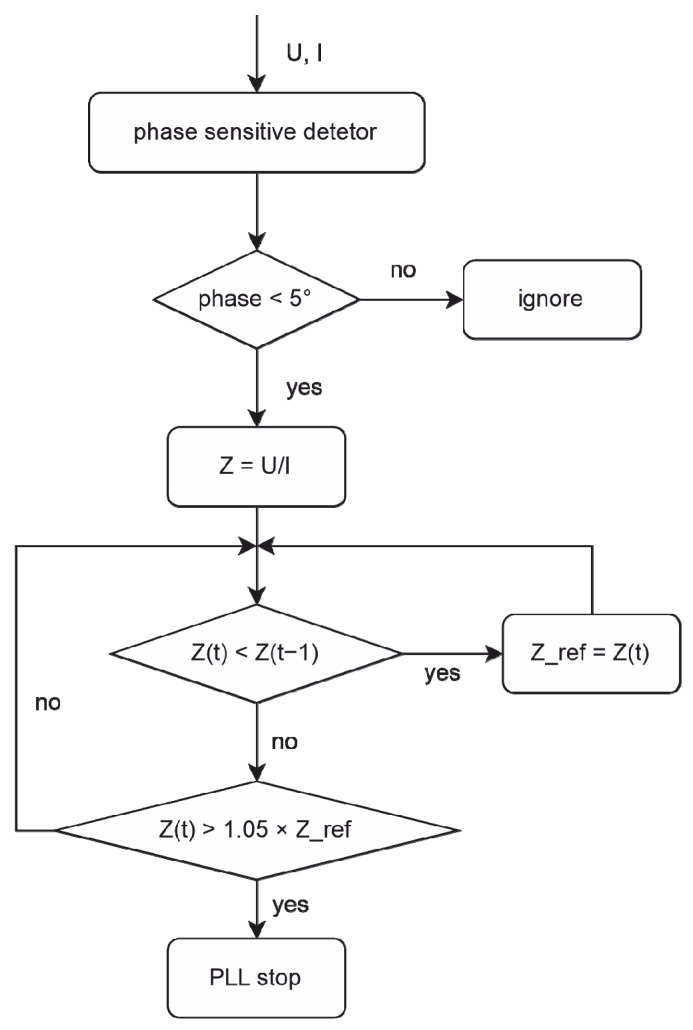
Flow chart of PLL-Controller with impedance monitoring.

In the investigation, a stop time was typically used to control the embossing, which may have resulted in different embossing depths and lowered the embossing quality. Precision control was needed, especially for stamps with a small size structure (a few hundred nanometers in depth direction). This could be performed in accordance with the impedance change that has been discussed above. The PLL controller calculated impedance every 2 ms. When the impedance exceeded a defined stop value (1.05× reference value), the controller terminated the signal and vibration stopped immediately. As a result, a ±2 ms precision control was achieved. Figure 7 depicts this specific control flow chart.

## 4. Results and Discussion

### 4.1. Embossing Time

As investigated in the preliminary study, impedance is a significant factor which can be used to effectively control the ultrasonic assisted embossing process. Thus, the impedance-based control strategy was applied in this work.

Table 2 shows the vibration amplitude of the ultrasonic transducer and the required embossing time at various output currents. It was noted that there was a threshold value (~2.7 A in this study) for ultrasonic assisted embossing. Below this value, the structure could not be transferred from the mold to the polymer even for a longer processing time. The reason was that when the amplitude of the vibration is small ($I_{\mathrm{output}}<2.7\;A$), the energy generated by the vibrations throughout the cycle time is insufficient due to the transmission being lost. As a consequence, the polymer did not receive enough power to heat up to the glass transition temperature and therefore remained in a glassy state.

The heating process (ultrasonic vibration) is greatly accelerated when the vibration amplitude is large. For example, when the vibration amplitude is 20 µm ($I_{\mathrm{output}}=2.7\;A$), it takes about 3 s to heat the contact area from room temperature to embossing temperature, whereas it takes about 0.5 s when the amplitude is 40.7 µm ($I_{\mathrm{output}}=5.5\;A$). When the vibration amplitude is too high, however, it has a detrimental impact on embossing quality, such as the appearance of bubbles or defects on the surface of some samples, a phenomenon known as ultrasonic cavitation.

To obtain a good balance of efficiency and embossing quality an output current of 4 A (vibration amplitude around 30 µm) was chosen for the investigation.

### 4.2. Determining the Period

**Figure 8:**
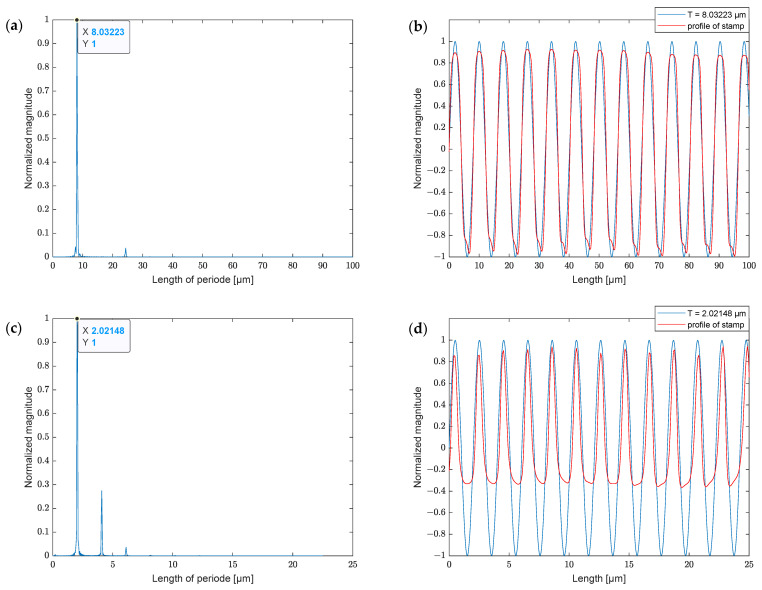
(**a**) Spectral density estimation of micrometric stamp; (**b**) comparison of cycle of micrometric stamp; (**c**) spectral density estimation of submicrometric stamp; (**d**) comparison of cycle of submicrometric stamp.

To determine the length period of the structures, a spectral density estimation was performed on the data collected by the microscope, since both of the aforementioned structures are periodic. The periods of the micrometric and the submicrometric structures are around 8 and 2 µm, as shown in Figure 8a,c, respectively. A comparison of the sine function and cross-sectional profiles is presented in Figure 8b,d, respectively.

### 4.3. Comparison of Embossing Quality with and without Impedance-Based Control

**Figure 9:**
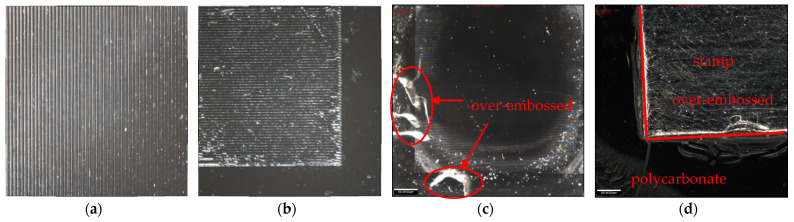
(**a**) embossing (vibration amplitude: 29.6 µm) with impedance-based control; (**b**) embossing (vibration amplitude: 40.7 µm) with impedance-based control; (**c**) over-embossed with impedance-based control; (**d**) over-embossed without impedance-based control.

**Figure 10:**
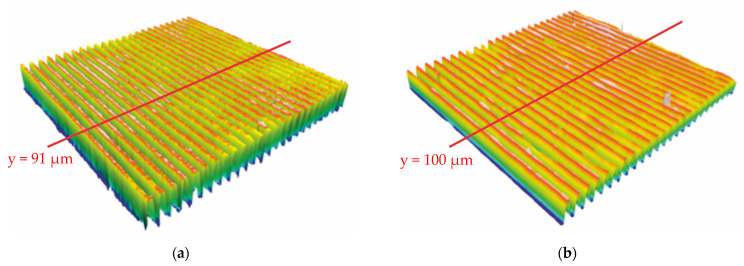
(**a**) Mold with micrometric structure; (**b**) embossed structure on polycarbonate.

**Figure 11:**
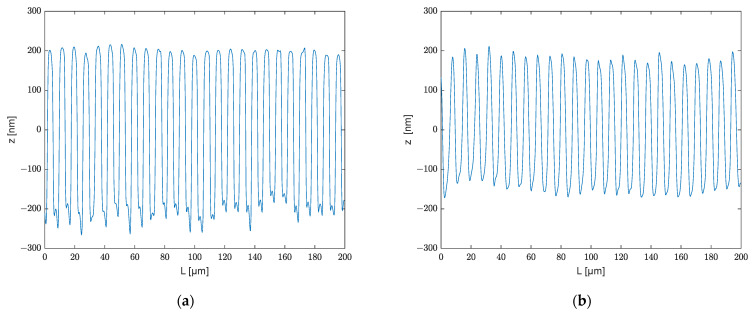
(**a**) Cross-sectional profile of mold at y = 91 µm; (**b**) cross-sectional profile of embossed polycarbonate at y = 100 μm.

A comparison of the embossing quality with and without impedance-based control is shown in Figure 9. The results shown in Figure 9a are from an ultrasonic amplitude of 29.6 µm, where distinct and flat grating lines can be seen (this result is consistent with the structures shown in Figure 10b and Figure 11b). The quality of the vibrations was not as satisfactory when the amplitude was too great (vibration amplitude: 40.7 µm, see Figure 9b). This is because when the polymer reached the embossing temperature, the amplitude remained large, making it difficult for the viscous fluid to enter the structural cavity. At the same time, some parts of the embossed samples had defects (as shown in Figure 9c), indicating that some over-embossing had occurred. It is possible for the stamp to be entirely embossed into the polymer if the impedance control method is not applied (as shown in Figure 9d). This is because when the temperature rises to embossing temperature at 30 µm, the amplitude is usually just around 1 s, after which the polymer will be in a viscous, rubbery condition and the temperature will not drop as quickly as the vibration is maintained. It will be easy to press the stamp into the polymer because it has been pre-pressured.

### 4.4. Results of Micrometric Structure

The average depth of embossing is another important parameter in determining embossing quality. The confocal microscope (MicroXAM-800) with 50× and 150× objectives was used to measure the structures.

With the 50× objective, the measurement window consisting of 724×724 pixels covered an area of 200×200 μm. The results are shown in Figure 10 and Figure 11. Figure 10a depicts the 3D structure of the mold and Figure 10b depicts the embossed polymer. Figure 11a,b show the cross-sectional profile of the mold as well as the polycarbonate at y = 91 μm and y = 100 μm, respectively, with the positions of the cross-section shown as red lines in Figure 10.

**Figure 12:**
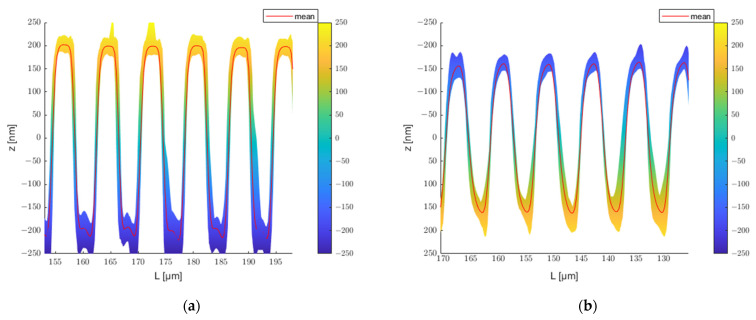
(**a**) Profiles in slice 153–198 μm; (**b**) Profiles in slice 125.5–170.5 μm.

Since the mold and embossed polymer have flipped shape contours, the profiles embossed on polycarbonate in Figure 11 have been flipped for a better comparison. Figure 12 exhibits the variety of profiles in a 45 µm slice of micrometer tool, as well as the polycarbonate embossed with it. The displayed ranges are 153–198 μm and 125.5–170.5 μm. The average depth is represented by the red line in the diagram. The contours in the figures indicate the error range of this grating line.

The overall shape of the embossed grating lines is obvious and uniform, as shown in Figure 10b and Figure 11b. The average height of the upper surface can approach 190 nm, implying that the viscous fluid can almost completely fill the groove. The lower surface has a lower average of 130 nm, which could be attributable to material shrinkage during the cooling phase. A suitable increase in the holding time may help to decrease shrinking.

### 4.5. Results of Submicrometric Structure

**Figure 13:**
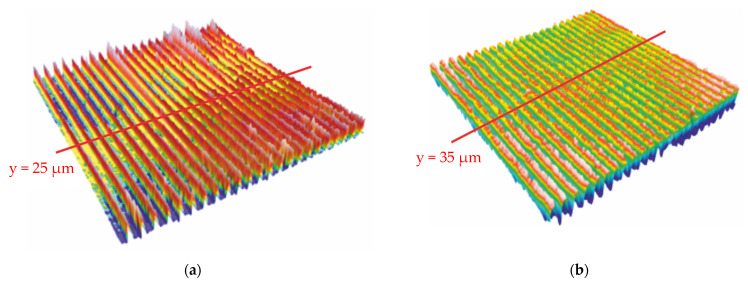
(**a**) Mold with submicrometric structure; (**b**) Embossed structure on polycarbonate.

**Figure 14:**
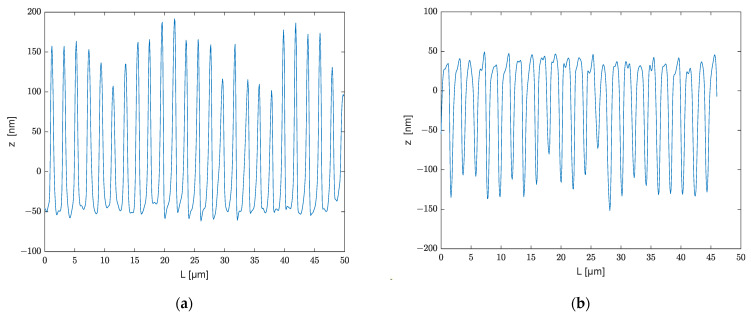
(**a**) Cross-sectional profile of submicrometric mold at y = 25 μm; (**b**) cross-sectional profile of embossed polycarbonate at y = 35 μm.

The results of the submicrometric structure with a measurement range of 46 × 46 μm, totally 508 × 508 pixels using the 150× objective, are shown in Figure 13, where Figure 13a depicts the structure of the mold and Figure 13b depicts the embossed polycarbonate. Figure 14a represents the profile of Figure 13a at 25 μm and Figure 13b provides the profile of Figure 13b at 35 μm. When comparing Figure 13a and Figure 13b, it can be observed that Figure 13a has more noise points than Figure 13b. There are two possible causes for these noise locations when collecting data from a microscope: One is that the mold has a sharper peak, making it more difficult to achieve an accurate value at that position using the confocal method. The second is the possible presence of dust at these locations during the measurement.

**Figure 15:**
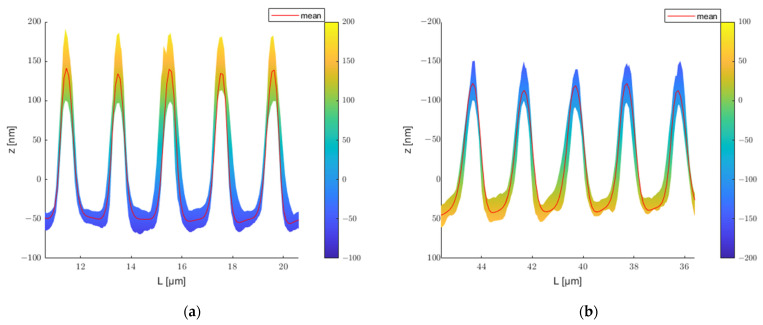
(**a**) Profiles in slice 10.6–20.6 μm; (**b**) profiles in slice 35.6–45.6 μm.

Figure 15 illustrates the range of profiles inside a 10 µm slice of a submicrometer tool and the embossed polycarbonate. The ranges are 10.6–20.6 μm and 35.6–45.6 μm, respectively. The average depth is denoted in the diagram by the red line. The error range of this grating line is indicated by the outlines in the figures.

The structures exhibited in Figure 13b and Figure 14b are of relatively poor quality in comparison to the micron structure in Figure 10b and Figure 11b. It is difficult to emboss grating lines with structures of tens to hundreds of nanometers. Figure 14b shows that the average height of the upper surface can reach 40 nm, which is similar to the depth of the lower surface in Figure 14a, suggesting that ultrasonic assisted embossing can potentially fill submicron grooves. However, there is a significant discrepancy on the bottom surface, with the better side reaching 150 nm and the poorer side barely reaching 50 nm. There are two likely causes for this: poor die quality of the stamp (see Figure 13a and Figure 14a) or material shrinkage during the cooling phase from embossing temperature to room temperature, which causes some of the lines to vanish.

### 4.6. Determining the Embossing Ratio

**Figure 16:**
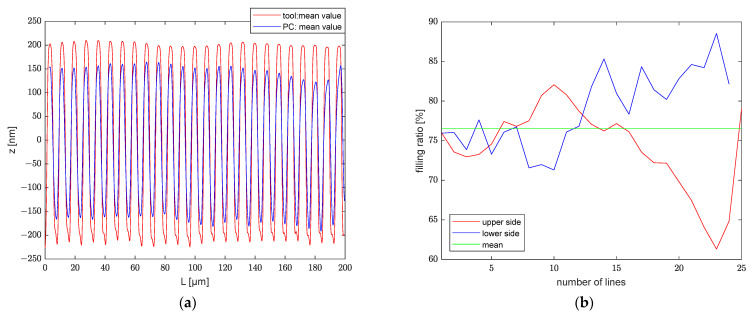
(**a**) Cross-sectional profile; (**b**) Filling ratio of lines.

**Figure 17:**
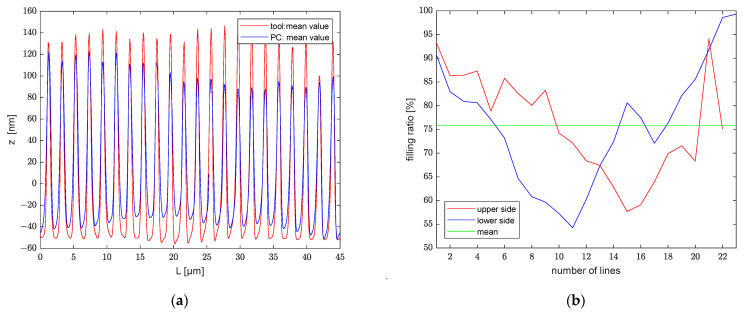
(**a**) Cross-sectional profile; (**b**) filling ratio of lines.

Table 3 shows the average and standard deviation of the depth for the two molds and the structures they embossed. The micron and submicron molds have depths of 419.39±5.48 nm and 188.40±10.76 nm, respectively; the transferred structures have depths of 320.80±6.78 nm and 141.61±12.96 nm, correspondingly. The average value of the cross-sectional profiles of the mold is represented by the red line in Figure 16a and Figure 17a, and the average value of the embossed structures is represented by the blue line. Figure 16b and Figure 17b show the analysis individual grating lines. The average transfer rate for both structures is greater than 75%. However, some distinctions exist between the grating lines. For the mold with micrometric structure, the filling rate for lines 1 to 17 is about average on both the top and bottom sides. In contrast, the filling rate for lines 18 to 24 on the bottom side is significantly higher than on the top side. As an example, line 24 has a filling rate of nearly 90% on the bottom side, while the rate on the top side is just greater than 60%. This indicates that the line pattern at that position is embossed deeper than the previous position, despite the fact that its average depth is not much different. The tool with a submicrometric structure has a larger tolerance, as illustrated in Figure 16b. The filling rate on both the top and bottom sides of the first line is greater than 90%, while the transfer rate is inferior for lines 10 to 15. There are several possible reasons for these differences: one is that the mold is shallower at that point. It could also be that the contact surface of the mold and the polymer are not perfectly horizontal.

## 5. Conclusions

In this study, an impedance-based control strategy was applied to control the ultrasonic assisted embossing process. The embossing quality was ensured by the fast response of the controller to the impedance change (2 ms), especially for the submicron structure. This control method can match the different stamp size and different ultrasonic vibration amplitudes. The temperature measurements show that the vibration significantly increased the temperature within the embossing region to above the glass transition temperature and the embossing was completed in less than a few seconds. Compared to traditional embossing process, less energy was consumed. Two types of embossing stamps (grating line width: 4 μm and 500 nm) were used in the experiments and both were able to obtain an average fill rate of more than 75%.

## Figures and Tables

**Table 1:** Ultrasonic assisted embossing (UAE).

Article	Structure and Dimension	Results	Process Time	Controlling Strategy
Chang et al. [15]	microlens arrays (ϕ250 µm; pitch: 400 µm)	max. height: 39.5 µm	<10 s	vibration time: 0.5, 1, 1.5 and 2 s hold time: 2, 4, 6, 8 s
Zhu el al. [16]	microcavities (272.4 × 500 × 500 µm)	max. depth: 271.7 µm max. replication rata: 99.74%	-	vibration time: 1, 1.5, 2 s hold time: 3.5, 4, 4.5 s
Runge et al. [17]	microfluidic device (groove: ϕ250 µm)	-	-	vibration time: 0.45, 1.05, 3.5 s hold time: 1, 1.05, 1.5 s
Kosloh et al. [18]	microfluidic channels (length: 1.6 cm; width: 1 mm; depth: 1 mm)	withstand a pressure difference of 700 kPa at a temperature of 220 °C	-	vibration time: 0.26, 0.28, 0.37, 0.5, 0.6, 3.2 s hold time: 1, 1.5 s
Qi et al. [19]	microgrooves (depth: 9.59 µm)	max. replication depth: 9.414 µm	<30 s	vibration time: 10, 15, 20, 25 s hold time: 0, 10, 20, 30 s
Luo et al. [20]	Grooves (min. width: 70 µm)	max. replication rate: 97.5%	<50 s	vibration time: 16, 19, 22 25 s hold time: 25 s
Cui et al. [21]	micro fluidic channels (min. width > 50 µm height < 0.5 cm)	-	-	vibration time: 200, 400 ms cooling time: several seconds
Zou et al. [22]	channels (length: 13.5 mm; width: 250 µm; depth: 100–500 µm)	height ratio: 58 ± 23% lateral shrinkage: 0.79 ± 0.17% (length) 0.76 ± 0.29% (depth)	0.7–3.3 s	-
Fan et al. [23]	grooves (width: 2 µm; depth: 200 nm)	replication height: ~12–~220 nm (PP) ~10–~230 nm (PMMA)	-	vibration time: 0.5, 0.7, 0.9, 1.2, 1.5 s hold time: 0.5, 0.7, 0.9, 1.2, 1.5 s

**Table 2:** Embossing time.

Output Current	Vibration Amplitude	Embossing Time ^1^
2.5 A	18.5 µm	∞
2.7 A	20.0 µm	~3.0 s
4.0 A	29.6 µm	~1.0 s
5.5 A	40.7 µm	~0.5 s

^1^ Ultrasonic vibrations time, time: t_1_–t_2_ in Figure 4.

**Table 3:** Mean depth and standard deviation of structures.

Units: nm	Micrometric		Submicrometric	
	Tool	Polycarbonate	Tool	Polycarbonate
Mean Depth	419.385	320.804	188.396	141.607
Standard Deviation	5.479	6.781	10.759	12.964

## Data Availability

The data presented in this study are available on request from the corresponding author.
